# The Nucleosome Remodeling and Deacetylase Complex NuRD Is Built from Preformed Catalytically Active Sub-modules

**DOI:** 10.1016/j.jmb.2016.04.025

**Published:** 2016-07-17

**Authors:** W. Zhang, A. Aubert, J.M. Gomez de Segura, M. Karuppasamy, S. Basu, A.S. Murthy, A. Diamante, T.A. Drury, J. Balmer, J. Cramard, A.A. Watson, D. Lando, S.F. Lee, M. Palayret, S.L. Kloet, A.H. Smits, M.J. Deery, M. Vermeulen, B. Hendrich, D. Klenerman, C. Schaffitzel, I. Berger, E.D. Laue

**Affiliations:** 1 Department of Biochemistry, University of Cambridge, 80 Tennis Court Road, Cambridge CB2 1GA, United Kingdom; 2 EMBL Grenoble, 71 avenue des Martyrs, CS 90181, 38042 Grenoble Cedex 9, France; 3 Wellcome Trust, Medical Research Council Stem Cell Institute, University of Cambridge, Gleeson Building, Tennis Court Road, Cambridge CB2 1QR, United Kingdom; 4 Department of Chemistry, University of Cambridge, Lensfield Road, Cambridge CB2 1EW, United Kingdom; 5 Department of Molecular Biology, Radboud Institute of Molecular Life Sciences, M850/3.79 Geert Grooteplein Zuid 30, 6525 GA Nijmegen, the Netherlands; 6 Cambridge Centre for Proteomics, Cambridge System Biology Centre, Wellcome Trust Stem Cell building, University of Cambridge, Department of Biochemistry, Tennis Court Road, Cambridge CB2 1QR, United Kingdom; 7 The School of Biochemistry, University of Bristol, University Walk, Clifton BS8 1TD, United Kingdom

**Keywords:** NuRD, nucleosome remodeling and deacetylase, ES, embryonic stem, MS, mass spectrometry, SEC, size exclusion chromatography, MALLS, multi-angle laser light scattering analysis, sptPALM, single-particle tracking photo-activated localization microscopy, mESCs, mouse ES cells, chromatin-remodeling, complex assembly, histone modification, nucleosome remodeling and deacetylase NuRD, transcription

## Abstract

The nucleosome remodeling deacetylase (NuRD) complex is a highly conserved regulator of chromatin structure and transcription. Structural studies have shed light on this and other chromatin modifying machines, but much less is known about how they assemble and whether stable and functional sub-modules exist that retain enzymatic activity. Purification of the endogenous *Drosophila* NuRD complex shows that it consists of a stable core of subunits, while others, in particular the chromatin remodeler CHD4, associate transiently. To dissect the assembly and activity of NuRD, we systematically produced all possible combinations of different components using the MultiBac system, and determined their activity and biophysical properties. We carried out single-molecule imaging of CHD4 in live mouse embryonic stem cells, in the presence and absence of one of core components (MBD3), to show how the core deacetylase and chromatin-remodeling sub-modules associate *in vivo*. Our experiments suggest a pathway for the assembly of NuRD *via* preformed and active sub-modules. These retain enzymatic activity and are present in both the nucleus and the cytosol, an outcome with important implications for understanding NuRD function.

## Highlights

• We have studied *Drosophila* nucleosome remodeling deacetylase (NuRD) assembly.
• NuRD consists of a core deacetylase complex, where MTA-like acts as the scaffold.
• This transiently associates with a chromatin remodeling sub-module including CHD4.
• Single-molecule imaging shows that the two sub-modules associate through MBD-like.
• NuRD comprises catalytically active sub-modules in both the cytosol and the nucleus.

## Graphical abstract

**Figure:**
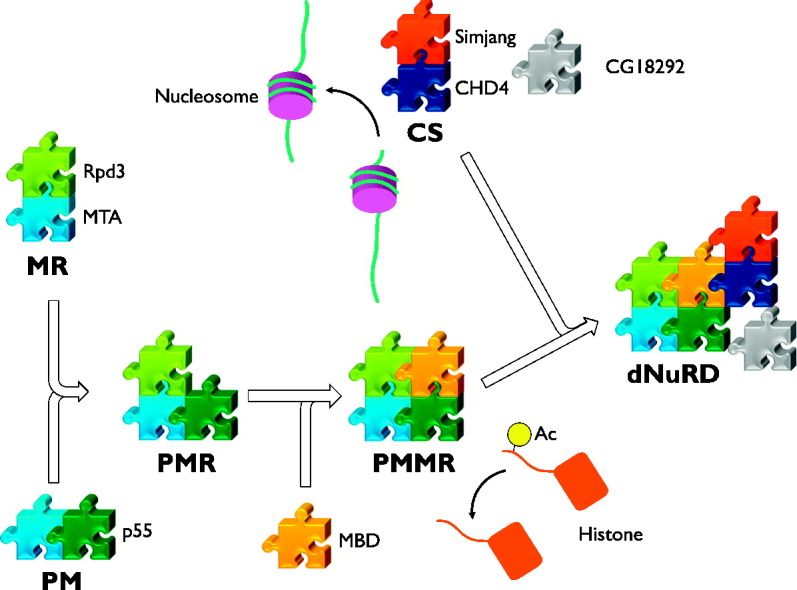

## Introduction

The nucleosome remodeling deacetylase (NuRD) complex combines chromatin remodeling and histone deacetylation activities [1], [2], [3], [4] and plays a central role in the differentiation of embryonic stem (ES) cells toward a committed lineage [5]. It has also been shown to either inhibit or promote the reprogramming of adult cells into induced pluripotent stem cells in different contexts [6], [7], [8] and to exert chromatin-independent functions [9]. In the absence of NuRD-mediated repression, pluripotency-associated genes are expressed at levels where ES cells are no longer able to respond to factors that induce differentiation [10]. Although the mechanisms are not yet clear, deacetylation of histone H3K27 by NuRD leads to recruitment of the polycomb repressive complex 2 resulting in subsequent H3K27 trimethylation at NuRD-controlled promoters [11].

In mammalian cells, the NuRD complex is known to comprise helicase-containing nucleosome remodeling ATPases (CHD3/4/5), histone deacetylases (HDAC1/2), histone chaperones (RbAp46/48), methyl-cytosine DNA-binding domain proteins (MBD2/3), metastasis tumor antigens (MTA1/2/3), and the GATA-type zinc finger proteins (GATAD2a/b) (for recent reviews, see refs. [12], [13]). [In *Drosophila*, the corresponding proteins are as follows: Mi-2 (CHD4), Rpd3 (HDAC1/2), p55 (RbAp46/48), MBD-like, MTA-like, and Simjang (GATAD2a/b). Note - we use CHD4 instead of Mi-2 throughout this paper.] It is therefore clear that considerable component variability exists within the mammalian NuRD complex suggesting that different versions of the complex might have distinct activities and biological functions in different cell types [14]. However, at present not enough is known about the assembly of the complex in order to predict which combinations of core components might form stable complexes—an essential prerequisite for understanding NuRD function.

Three structures provide crucial information about interactions within the NuRD complex. Firstly, the structure of the ELM2–SANT domain of MTA1 bound to HDAC1 shows that MTA1 dimerises to create a 2:2 (MTA1:HDAC1) complex [15]. Secondly, the structure of a RbAp48–MTA1 complex has shown that a C-terminal sequence motif in MTA1 is sufficient to recruit RbAp48 [16]. Finally, the structure of a coiled-coil formed between MBD2 and GATAD2A [17] provides a third interaction. There are also structures of isolated domains, including the PHD domains [18] and the chromodomains (PDB code: 4O9I) of CHD4, the methyl-DNA binding domain of MBD2 [19], and the SANT domain of MTA3 (PDB code: 2CRG).

To investigate NuRD assembly, we studied the *Drosophila* complex, where there is only a single gene for each major NuRD component. We exploited label-free quantitative mass spectrometry (MS)-based proteomics of purified endogenous complexes, systematic co-expression/purification of NuRD components in insect cells using the MultiBac system, and biochemical and biophysical characterization of the sub-modules.

## Results

### Purification of *Drosophila* NuRD reveals a putative core complex

**Fig. 1:**
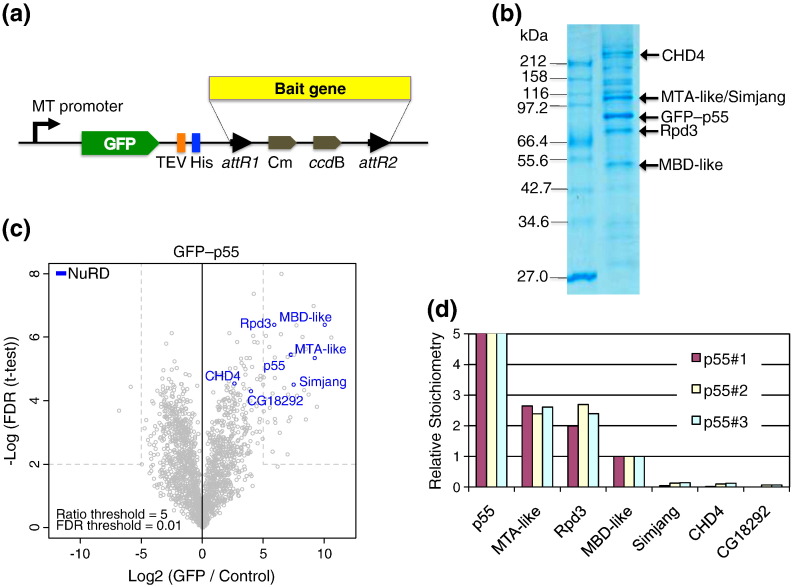
Endogenous dNuRD complex from *Drosophila.* (a) The endogenous dNuRD complex was purified from *Drosophila* S2 Schneider cells using affinity capture of a GFP-tagged bait protein. The construct used for expressing tandem affinity purification (TAP) tagged bait protein is shown in a schematic fashion. MT, matellothionin promoter; GFP, green fluorescent protein; TEV, tobacco etch virus NIa protease recognition sequence; His, oligohistidine tag; attR1/attR2, recombination sites; Cm, chloramphenicol resistance marker; ccdB, selection cassette. (b) The proteins captured by GFP-trap resin (Chromotek) from a nuclear extract of cells expressing GFP-p55 were analyzed by SDS-PAGE and Coommassie brilliant blue (CBB) staining. Components identified by MS and Western blot are marked with arrows and denoted. (c) Statistically enriched proteins in the GFP-p55 pull-down experiment were identified in a permutation-based FDR-corrected *t* test and are shown in a Volcano plot. (d) The relative stoichiometries of components within endogenous dNuRD were determined using intensity-based absolute quantification [23]. The values are normalized to MBD-like (stoichiometry = 1.0). Three individually purified samples (denoted p55#1, p55#2, and p55#3) consistently indicate a ratio of 2: 2: 1 for the NuRD core components MTA-like, Rpd3, and MBD-like. In contrast, CHD4, Simjang, and CG18292 appear to bind sub-stoichiometrically.

We employed a GFP-tagging approach [20], [21] to purify the NuRD complex from *Drosophila* S2 Schneider cells. We modified an existing Gateway plasmid with a metallothionein promoter-driven expression cassette so that it expressed proteins with a GFP–TEV_x2_–histidine_x10_ tandem affinity purification tag (Fig. 1a). We expressed the tagged NuRD subunit p55 and generated stable *Drosophila* S2 Schneider cell line, taking particular care to ensure that the level of heterologous expression matched that of endogenous p55 as judged by Western blot. We affinity purified complexes containing tagged p55 (Fig. 1b) and analyzed the proteins by MS (Fig. 1c) revealing the presence of known NuRD complex components p55 (the bait), MTA-like, Rpd3, MBD-like, Simjang, CHD4, and CG18292 (the homolog of human DOC1 [22]). In addition to these NuRD-specific proteins, subunits of other complexes containing p55 were also detected (Supplementary Fig. S1). We used label-free quantitative MS-based proteomics to estimate the stoichiometry of individual subunits [23], [24]. Our analyses consistently revealed a bipartite distribution of NuRD subunits. MTA-like, Rpd3, MBD-like, and p55 were present in one to several copies (Fig. 1d), while Simjang, CHD4, and CG18292 were sub-stoichiometric. The ratio of MTA-like, Rpd3, and MBD-like was 2:2:1, while p55 (the bait) was present in considerable excess consistent with its presence in multiple chromatin complexes (Supplementary Fig. S1). In summary, the majority of the captured material contained only a subset of components, possibly representing a physiological NuRD core complex. Of note, we could purify substantial material from cytosolic extracts, indicating that NuRD and its sub-modules are not confined to the nucleus.

### Recombinant expression produces a stable core NuRD complex

**Fig. 2:**
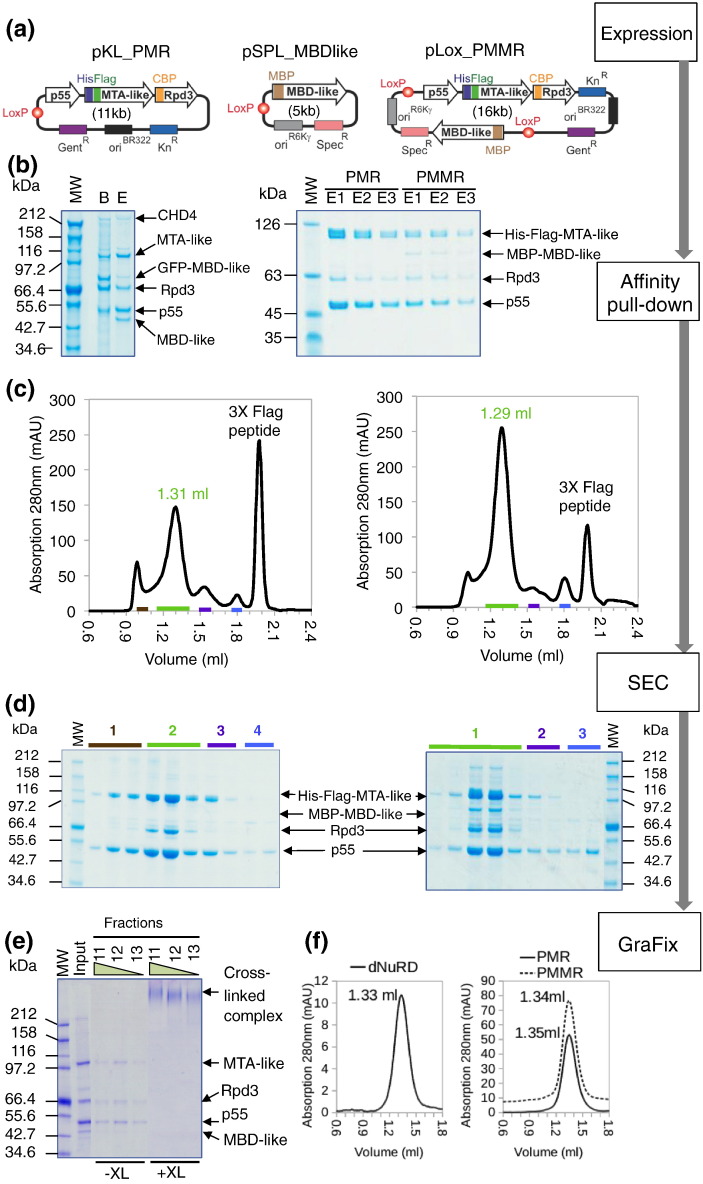
Recombinant NuRD complexes. (a) Constructs used to express recombinant NuRD complexes with the MultiBac system [25] are shown in a schematic representation. pKL and pSPL are MultiBac Acceptor and Donor plasmids, respectively [25]. LoxP sites used for Cre recombination are shown as circles filled in red. Resistance markers and origins of replication are depicted as colored boxes. Genes encoding NuRD subunits are shown as arrows filled in white. PMR stands for p55, MTA-like, and Rpd3, respectively. Tags are shown as colored rectangles and marked. His-Flag, tandem affinity purification tag comprising a deca-histidine tag and a triple FLAG epitope (DYKDHDGDYKDHDIDYKDDDDK); MBP, maltose binding protein; CBP, calmodulin binding peptide. Gent, gentamycin resistance marker; Kn, kanamycin resistance marker; Spec, spectinomycin resistance marker. Origins of replication (ori^BR322^, ori^R6Kγ^) on pKL and pSPL, respectively, are indicated. pLox_PMMR is generated from pKL_PMR and pSPL_MBD-like by Cre-LoxP-mediated plasmid fusion. PMMR contains in addition a gene encoding for MBP-tagged MBD-like protein. The size of the plasmids (in kilobases, kb) is indicated in brackets. (b) SDS-PAGE section showing endogenous NuRD complex purified by affinity capture using GFP trap (left). Four subunits (MTA-like, MBD-like, Rpd3 and p55 appear as dominant Coomassie stained bands in SDS-PAGE gels (marked by arrows). A weak Coomassie stained band corresponding to CHD4 is labeled. MW, molecular weight marker; B, Complex bound to GFP-nanobody beads; E, Complex eluted after TEV cleavage. Molecular masses of marker bands are indicated in kilodalton (kDa). SDS-PAGE sections of MultiBac-produced recombinant PMR and PMMR complexes are shown on the right. Subunits are marked by arrows and labeled. E1-3 denotes stepwise batch elution fractions from anti-FLAG beads. (c) SEC profiles and corresponding SDS-PAGE gel sections are shown for purified recombinant PMR complex (left) and purified recombinant PMMR complex (right). Elution fractions are marked by colored bars in the SEC profiles (top) and above the SDS-PAGE sections in (d) below. The peaks in the SEC profiles containing PMR or PMMR complex are highlighted by a number (in green) representing the retention volume. The peak at around 2-ml retention volume contains Flag peptide from the affinity purification step. Both the PMR and PMMR complexes elute at around 1.3 ml, indicative of similar hydrodynamic radii. The molecular mass of PMMR was determined by SEC-MALLS and corresponds to 600 kDa (Supplementary Fig. S2). (e) NuRD complexes were further purified by gradient centrifugation in the presence and absence of glutaraldehyde cross-linker (GraFix). Peak fractions of the gradients were analyzed by SDS-PAGE with uncross-linked (− XL) and cross-linked (+ XL) samples loaded side-by-side (left). The peak fractions from the + XL gradient contain a single high-molecular weight band representing cross-linked complex. (f) SEC analyses of GraFIX-treated endogenous NuRD prepared by GFP-trap affinity capture (dNuRD, left) as well as recombinant PMR and recombinant PMMR (right) are shown. All complexes elute at around 1.3-ml retention volume indicating comparable overall molecular dimensions.

We next produced a recombinant core NuRD complex, formed by p55, MTA-like, MBD-like, and Rpd3 (PMMR), using the MultiBac insect cell expression system [25]. A transfer construct encoding p55, MTA-like, and Rpd3 (pKL_PMR) was fused to a construct encoding MBD-like (pSPL_MBDlike) by Cre-LoxP-mediated plasmid fusion yielding pLox-PMMR, which encodes all four subunits of the putative NuRD core complex (Fig. 2a). pKL_PMR and pLox_PMMR were expressed in Sf21 insect cells and resulted in stable PMR and PMMR complexes (Fig. 2b, right panel). Both were found in distinct cytosolic and nuclear pools, as with the endogenous preparations. In parallel, we purified the endogenous complex by tagging MBD-like, resulting in dNuRD containing p55, MTA-like, MBD-like, and Rpd3—CHD4, Simjang, and CG18292 were again lost during the purification (Fig. 2b, left panel) indicating a transient association. We observed minor differences between the recombinant material and the endogenous preparation for MTA-like and Rpd3, due to minor proteolysis or the presence of different levels of post-translational modifications, respectively.

The recombinant PMR and PMMR complexes elute at similar retention volumes by size exclusion chromatography (SEC) showing that the particles have comparable dimensions (Fig. 2c). In addition, both profiles showed smaller peaks containing only p55 and MTA-like (PM). The endogenous dNuRD, as well as recombinant PMR and PMMR, was subjected to gradient centrifugation in the presence of mildly cross-linking glutaraldehyde [26] followed by SEC (Fig. 2e and f). The three complexes eluted in a single symmetric peak with similar retention volumes (Fig. 2f), confirming that all three assemblies have comparable molecular dimensions. SEC of the PMMR complex followed by multi-angle laser light scattering analysis (SEC-MALLS) indicated a molecular mass of ~ 600 kDa (Supplementary Fig. S2). To corroborate our observations, we co-expressed all seven NuRD subunits detected in our GFP-affinity capture experiment (Fig. 1d). This too resulted in purification of the PMMR complex, consistent with our preparations of the endogenous complex (Supplementary Fig. S3).

### EM of dNuRD, PMMR, and PMR suggests that they have similar shapes

**Fig. 3:**
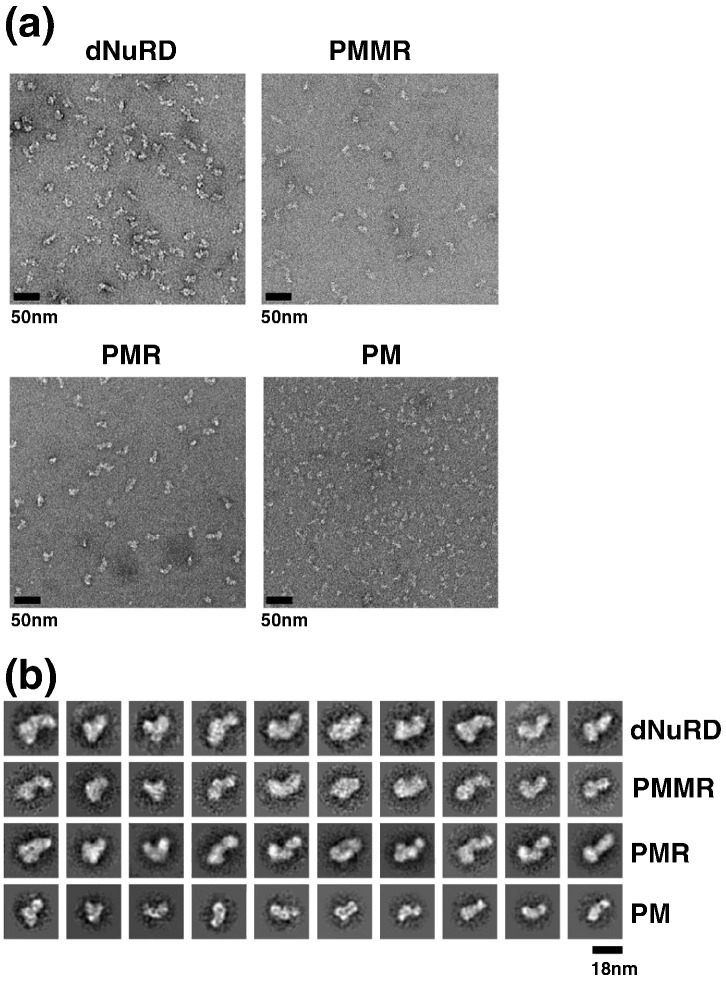
Electron microscopy of NuRD complexes. (a) Representative negative-stain electron micrographs are shown of purified endogenous dNuRD (top, left) and recombinant PMMR (top, right), PMR (bottom, left), and PM (bottom, right) complexes stained with uranyl acetate. The scale bar corresponds to 50 nm. (b) Multi-variance statistical analysis using IMAGIC-5 was used to obtain reference-free 2D class averages of the dNuRD, PMMR, PMR, and PM complexes. Class averages with a similar orientation in the dNuRD, PMMR, and PMR samples indicate the presence of particles with similar shape and comparable dimensions. In contrast, considerably smaller particles are observed in the PM sample. The scale bar corresponds to 18 nm.

Uranyl-acetate stained micrographs revealed homogenous particles for endogenous dNuRD and recombinant PMMR and PMR complexes with similar shapes and dimensions, confirming our previous findings (Fig. 3a). Particles were picked and 2D class averages computed by reference-free multivariate statistical analysis using EMAN2 and IMAGIC-5 [27], [28]. Comparison of representative class averages from the dNuRD, PMMR, and PMR data clearly shows the presence of similarly shaped, homogenously structured particles, consistent with our biochemical and MS analyses which suggest that the core NuRD complex comprises stably associated p55, MTA-like, MBD-like, and Rpd3 proteins. The 2D multivariate statistical analyses show that the PM complex, by contrast, forms significantly smaller particles.

### dNuRD, PMR, and PMMR all deacetylate comparably

**Fig. 4:**
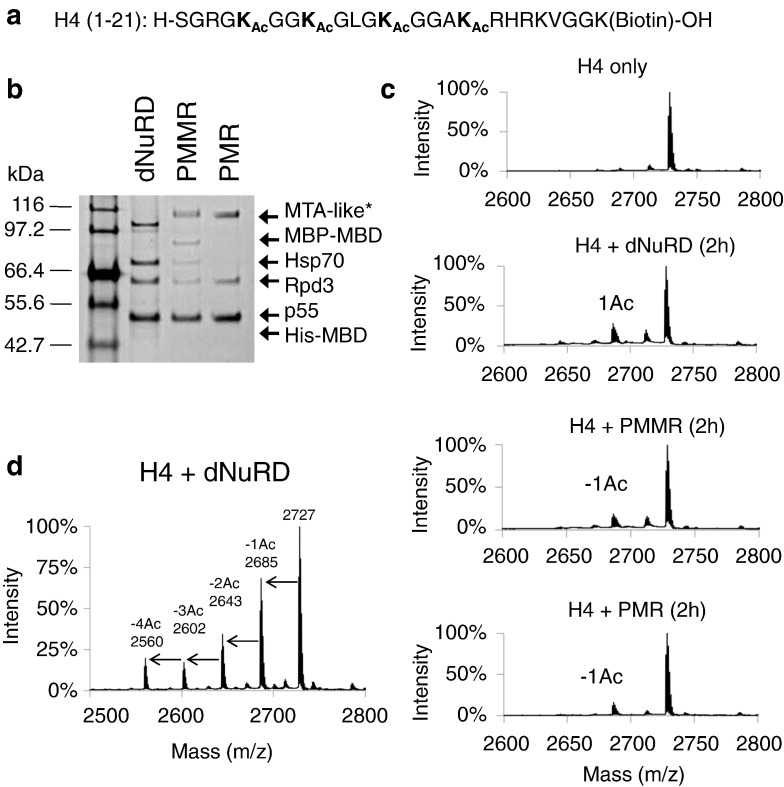
Deacetylase activity of NuRD complexes. (a) A synthetic peptide corresponding to the N-terminal 21 amino acids of histone H4 was used as substrate for the deacetylation assays. The amino acid sequence is shown with acetylated lysine residues indicated (5, 8, 12, and 16; Ac, acetylated lysine ε-amino group). A biotin tag is present at the C-terminus. (b) SDS-PAGE section from a NuPAGE Bis-Tris gradient mini gel (4%–12%) showing that the concentration of endogenous (dNuRD) and recombinantly produced PMR and PMMR complexes is similar in all the assays. Recombinant MTA-like protein with the oligohistidine-triple FLAG epitope tandem affinity purification tag (marked with an asterisk) migrates at a higher molecular weight than endogenous MTA-like. A minor contaminant in the PMMR preparation was identified as Hsp70. (c) The deacetylase activity of dNuRD, PMR and PMMR complexes was assessed by MALDI-TOF MS analysis of the acetylated peptide substrate. Deacetylase reactions were terminated by addition of acid after 2 h incubation and analyzed by MALDI-TOF. H4, tetra-acetylated histone H4 (1–21) peptide; − 1Ac, H4 (1–21) peptide with one acetyl group removed. (d) MALDI-TOF analysis after an overnight deacetylation reaction of tetra-acetylated histone H4 (1–21) peptide by the dNuRD complex. Substrate peptides lacking up to four acetyl groups (complete deacetylation) are detected.

We asked whether the deacetylase activity of Rpd3 [29] is different in endogenous dNuRD, and the recombinant PMR and PMMR complexes using a synthetic N-terminal H4 peptide acetylated at lysines K5, K8, K12, and K16 (Fig. 4a). When the H4 peptide was incubated for 2 h in the presence of equal amounts of dNuRD, PMR, or PMMR complex (Fig. 4b), the removal of one acetyl group was detected by MS analysis, with comparable intensity in all the different samples (Fig. 4c). Thus, the absence of MBD-like does not influence Rpd3 activity. Addition of inositol (1,4,5,6) tetra-phosphate [15] had no effect on deacetylation, suggesting that this co-factor remains stably bound in our preparations. Longer reactions resulted in multiple lysine residues being deacetylated (Fig. 4d), confirming Rpd3's broad substrate specificity [29].

### Interaction of CHD4 with the core PMMR complex

We tested whether we could detect weak CHD4/PMMR interactions in electrophoretic mobility shift assays with nucleosomes having been unable to demonstrate an interaction between CHD4 and the core PMMR complex only. However, while robust interactions between CHD4 and nucleosomes were detected, no additional complex was formed when we added the PMMR complex (Supplementary Fig. S4), suggesting that other components are needed for the assembly of intact NuRD.

**Fig. 5:**
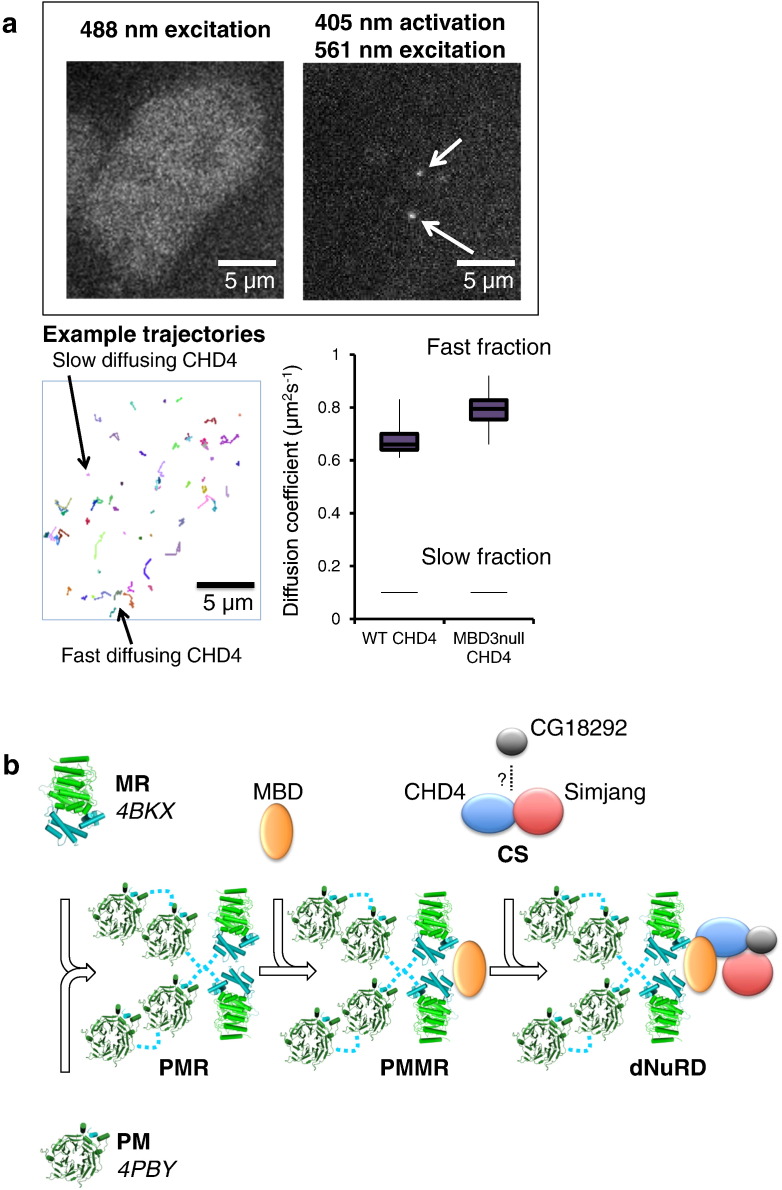
Interaction of CHD4 with the core PMMR complex. (a) 2D single-molecule tracking of single mEos3-tagged CHD4 molecules in live mESCs show differences in the diffusion of a sub-population of CHD4 molecules in the presence (WT) and absence of MBD3 (MBD3-null). Representative images of the same cell are shown (top right) using low power 488-nm excitation (green form of mEos3) and 405-nm/561-nm excitation (photo-activated red form of mEos3). A small number of the individual tracks from this cell are also shown indicating the fast and slow diffusing fractions of CHD4. The exact diffusion coefficients extracted from the data are shown in a box-and-whisker plot (lower right). A total of 23,854 and 20,039 tracks were analyzed for the wild-type and MBD3-null cells, respectively. (b) A putative model of NuRD complex assembly is shown in a schematic representation. Protein interactions which can be modeled from known molecular structures (PDB identifiers 4BKX and 4PBY) are shown. The interactions between MTA-like (light blue) and Rpd3 (light green) (MR) are based on the structure of MTA1/HDAC1 elucidated by X-ray crystallography (PDB ID 4BKX, [15]). The interaction of p55 (dark green) with MTA-like is modeled on the structure of RbAp48 with a C-terminal fragment of MTA1 (PDB ID 4PBY, [16]). Two molecules of p55 interact with one molecule of MTA-like *via* interactions with two related peptide motifs in the centre and C-terminus of MTA-like (residues 618–622 KKAARQ and 848–853 RRAARK, respectively). The p55, MTA-like, and Rpd3 proteins assemble into a complex with presumed 4:2:2 stoichiometry (PMR). One copy of MBD-like protein (yellow) interacts with PMR to yield the core NuRD complex (PMMR) with a stoichiometry of 4:2:1:2 consistent with that determined by SEC-MALLS (Supplementary Fig. S2). Simjang (dark blue), CHD4 (red), and CG18292 (putative Doc1, gray) are more loosely associated, peripheral subunits that interact with the core NuRD complex. CHD4 does not interact with core PMMR by itself, but likely interacts as a sub-module with Simjang (and perhaps CG18292) *via* the interaction of Simjang with MBD-like (as structurally characterized by the p66α–MBD2 interaction), to give holo-NuRD. The interaction of the two sub-modules combines the ATP-dependent chromatin remodeling function of CHD4 with the deacetylase activity conferred by Rpd3 in one holo-enzyme. Preformed PMR and PMMR sub-modules (and quite possibly MR) represent stable and enzymatically active deacetylases, which may catalyze distinct cellular functions on their own.

Because we were unable to purify either Simjang or CG18292 sufficiently for reconstitution with CHD4 and the core PMMR complex, we turned to single-particle tracking photo-activated localization microscopy (sptPALM) to study the interaction *in vivo*. We used mouse ES cells (mESCs) and generated knock-in cell lines where we tagged CHD4 with the photo-activatable mEos3 protein [30]. We then measured CHD4 dynamics using sptPALM at 10-ms time resolution [31] in both wild-type and MBD3-null cells [32]. (MBD3 is the major mouse homolog of MBD-like in mESCs.) The fluorescence trajectory from an individual mEos3 molecule could be tracked, with a mean trajectory length of 5.1 ± 0.3 frames, and we recorded an average of ~ 3000 trajectories per movie of one or two cells. Jump-distance analysis showed that in wild-type mESCs, CHD4 exhibits two major diffusion coefficients (0.100 ± 0.008 and 0.65 ± 0.04 μm^2^ s^− 1^). The slowly diffusing fraction 49% ± 6% appears to be stably bound to chromatin, as its diffusion is similar to that observed for histone H2B [31]. [The value is limited by the precision to which a single molecule's position can be determined, which in our experiments is 62 ± 4 nm corresponding to a diffusion coefficient of ~ 0.1 μm^2^ s^− 1^ at 10-ms time resolution (as confirmed in fixed cells).] In MBD3-null cells, CHD4 still exhibits the slowly diffusing fraction (49% ± 3%; 0.100 ± 0.008 μm^2^ s^− 1^), but the fast moving fraction now moves significantly more quickly (0.80 ± 0.08 μm^2^ s^− 1^) (*p* < 0.004). This is consistent with our hypothesis that CHD4 should move more rapidly as the smaller CS complex in MBD3-null cells, while in wild-type cells, it would diffuse more slowly because it is associated with PMMR (Fig. 5b).

## Discussion

Here, we report our dissection of NuRD, a transcriptional regulator that combines deacetylase and ATP-dependent chromatin remodeling activities. We purified endogenous NuRD from *Drosophila* S2 Schneider cells and found that the complex we purified consists of only a subset of its constituent proteins (p55, MTA-like, MBD-like, and the deacetylase Rpd3) with a well-defined stoichiometry. The three remaining subunits, Simjang, the ATP-dependent helicase CHD4, and the *Drosophila* DOC1 homolog CG18292, in contrast, appear to be peripheral, more loosely attached NuRD subunits which are lost during purification. Reminiscent of what we found for TFIID [33], our results are consistent with a bipartite architecture of NuRD in which PMMR interacts with Simjang, CHD4, and Doc1 at certain times to yield a bi-functional holo-NuRD complex, which combines the two known enzymatic activities (histone deacetylation and chromatin remodeling).

We applied a two-pronged approach to verify our hypothesis. First, we further purified the endogenous dNuRD complex. Second, we co-expressed the four putative core subunits using the MultiBac system [25] and confirmed that the endogenous dNuRD and recombinant PMMR complexes contained the same four constituent proteins. SEC-MALLS defined the stoichiometry of PMMR as 4:2:2:1 for p55, MTA-like, Rpd3, and MBD-like in good agreement with our proteomics results. In support of this overall stoichiometry we found that the RbAp proteins also bind to a homologous central sequence in the MTA proteins in addition to binding to the C-terminus^16^ (data not shown). We conclude that PMMR may indeed represent a core NuRD complex. We also expressed recombinant PMR complex, which eluted at similar SEC retention volumes as PMMR. All three complexes, endogenous dNuRD and recombinant PMMR and PMR, exhibited virtually identical deacetylase activities, but are devoid of the chromatin remodeling activity conferred by CHD4. Of note, in all three, we observed distinct, comparably prominent, cytosolic, and nuclear pools. The presence of these cytosolic pools hints at non-nuclear functions of NuRD. Analysis of our complexes, recombinant and endogenous, by negative stain electron microscopy compellingly underscored our results, evidencing homogenous particles of similar size and shape for PMR, PMMR, and the endogenous dNuRD complex. By contrast, the PM complex resulted in significantly smaller-sized particles. We conclude that partial NuRD complexes can be produced that retain deacetylase activity conferred by Rpd3, in the absence or presence of MBD-like. PMR, in turn, is assembled from the binary complexes PM and MR, the latter of which contains the histone deacetylase. Previous studies have characterized the PM and MR interactions at near-atomic resolution [15], [16], providing invaluable molecular-level insight about key interactions in the NuRD assembly pathway to the holo-complex (see Fig. 5b).

Our results imply that the putative PMMR core NuRD complex then accretes CG18292, CHD4 and Simjang, possibly when triggered by cellular or external stimuli, to give rise to holo-NuRD. We were unable to demonstrate an interaction between PMMR and CHD4, which we and others [34], [35], [36] have shown functions as an ATPase-dependent remodeler by itself. However, our previous chemical cross-linking/MS experiments [37] show that, in the mammalian NuRD complex, CHD4 and GATA2Da associate. We therefore hypothesized that the holo-NuRD complex may assemble through the interaction of a chromatin-remodeling sub-module, comprising CHD4 and Simjang (CS) with PMMR. This interaction between Simjang in CS and the MBD-like subunit in PMMR would occur through the formation of a coiled coil, as seen in the structure of the GATA2Da–MBD2 complex [17].

We sought evidence for the hypothesis that a Simjang/MBD-like (or GATA2Da/b–MBD2/3) interaction is important for the association of the core PMMR deacetylase and CS chromatin-remodeling sub-modules, by carrying out single molecule tracking studies of CHD4 in the presence and absence of MBD3 in mESCs. The slowly moving molecules are not affected by the presence or absence of MBD3, and given the higher concentrations of CHD4 in the cell, and its known NuRD-independent functions, we suspect that they may correspond to CHD4 that is not associated with NuRD [38]. The more rapidly moving CHD4 molecules (which we expect are diffusing along chromatin) provide direct evidence that the MBD component plays an important role in the association of CHD4 with NuRD, consistent with our hypothesis that the CS and PMMR sub-modules interact *via* a Simjang/MBD-like interaction (see Fig. 5b). In this model, CG18292 (or DOC1 in mammalian cells) might either interact with PMMR by itself or as part of the CS complex.

In summary, our experiments suggest a pathway for the assembly of the complete and functional NuRD complex *via* preformed and active sub-modules. The fact that the sub-modules retain similar enzymatic activities and are present in different cellular compartments when expressed at endogenous levels is consistent with the idea that they may have independent activities in both the nucleus and the cytosol.

## Materials and Methods

### Production and purification of NuRD complexes

Endogenous NuRD complex was purified from *Drosophila* S2 Schneider cells using stable cell lines and a metallothionein-induceable promoter (Thermo Fischer Scientific) as described in Supplementary Data. Recombinant NuRD complexes were overexpressed using the MultiBac system [25] and purified as detailed in the Supplementary Methods.

### Mass spectroscopy

The generation of the GFP-tagged p55 and GFP control S2 Schneider cell lines has been described previously [16]. Sample cleanup and nanoLC-MS/MS analysis was also carried out as described [18]. Statistically enriched proteins in the GFP-p55 pull-down were identified by a permutation-based FDR-corrected *t* test. The label-free quantification intensity of the GFP pull-down relative to the control pull-down, GFP only (fold change, *x*-axis) is plotted against the − log10-transformed *p* value of the *t* test (*y*-axis). The proteins in the upper right corner represent the bait and its interactors. The relative stoichiometry of the endogenous dNuRD components was determined using the intensity-based absolute quantification method [18].

### Electron microscopy

For the endogenous dNuRD complex, 5 μl was adsorbed onto carbon film for 60 s followed by 2% uranyl acetate staining for 30 s. One hundred micrographs were recorded under low-dose conditions at room temperature on a F20 electron microscope (FEI) operating at 200 kV using a 4k × 4k CCD camera. The nominal magnification used was 40,000, which corresponds to a pixel size of 2.855 Å.

SEC analysis of both recombinant PMMR and PMR complexes revealed the presence of an additional smaller complex comprising only MTA-like and p55 (PM) eluting in a separate peak. Five microliters of PMMR, PMR, or PM was adsorbed onto carbon film and stained as described above for the endogenous dNuRD sample. Low-dose images of 320, 150, and 120, respectively, were recorded with a JEOL 1200 EX II microscope operated at 100 kV using a 2k × 2k CCD camera at a nominal magnification of 40,000. All micrographs were corrected for the contrast transfer function and phase-flipped using the program bctf from Bsoft [39]. A total of 14,288 (dNuRD), 4753 (PMMR), 3610 (PMR), and 7901 (PM) particles were picked and extracted manually using e2boxer.py from EMAN2 [27]. Picked particles were subjected to four rounds of 2D multi-variance statistical analysis and classification using IMAGIC-5 [28], resulting in 200 reference-free classes for the dNuRD and PMMR complexes, and 100 reference-free classes for the PMR and PM complexes.

### Deacetylase activity assay

One microgram each of endogenous dNuRD, recombinant PMR, or recombinant PMMR complex was mixed with 20 μM of a synthetic tetra-acetylated peptide corresponding to residues 1 to 21 of histone H4 (AnaSpec, AS-64,989) in deacetylation buffer [25 mM Hepes (pH 7.5), 200 mM NaCl, 0.1 mM EDTA, and 2% glycerol supplemented with fresh 0.5 mM DTT]. Protein complex concentrations were confirmed by SDS-PAGE using a 4%–12% NUPAGE® Bis-Tris gradient mini gel (Invitrogen) stained with SYPRO® Ruby Protein Gel Stain (Molecular Probes™). A 20-μL deacetylation reaction was incubated at 30 °C and stopped after 2 h by adding 5 μL of Stop Solution (1 M HCl and 0.16 M acetic acid). Reaction mixtures were analyzed by MALDI-TOF MS.

### sptPALM

mESCs expressing CHD4 tagged at the C-terminus with mEos3 [30] were generated as described in Supplementary Data. Prior to imaging, cells were washed once with PBS and then cultured for a day in phenol-free serum and LIF conditions. For cell fixation, cells were incubated in 1% formaldehyde in PBS at room temperature for 15 min. The fixed cells were washed with PBS before imaging. Collimated 561 nm (Cobolt, Jive 200), 488 nm (Toptica, iBeam Smart 488,100 mW), and 405 nm (Oxxius, LaserBoxx 405) laser beams were aligned and focussed at the back aperture of an Olympus 1.49 NA 60 × oil objective mounted on an IX71 Olympus inverted microscope frame. The power of the collimated beams at the back aperture of the microscope was, respectively, 10 kW/cm^2^, 1 kW/cm^2^, and 10–100 W/cm^2^. The fluorescent signal was filtered with a four-band dichroic (Semrock, Di01-R405/488/561/635) and either a 488 long-pass filter (Semrock, BLP01-488R, for beads), or a combination of a 561 long-pass (Semrock, BLP01-561R) and a 587 band-pass filter (Semrock, FF01-587/35), expanded through a 2.5 × achromatic beam expander (Olympus, PE 2.5 × 125) and finally projected onto an EMCCD (Photometrics, Evolve 512). Image stacks of 10,000 frames were collected and then analyzed by software that detects single-molecule trajectories from the PALM movies and carries out the jump distance analysis [40]. Only fluorescent puncta smaller than 5 pixels and with a signal-to-noise greater than 4 were analyzed. Fluorescent puncta were considered to be the same molecule if they were within 5 pixels between frames because we do not expect to see diffusion coefficients greater than 30 μm^2^ s^− 1^ for CHD4.

## Appendix A Supplementary data

Supplementary material Image 2
