# Supplementary material for: The Nucleosome Remodeling and Deacetylase Complex NuRD Is Built from Preformed Catalytically Active Sub-modules
Source: J Mol Biol. 2016 Jul 17;428(14):2931–42. doi: 10.1016/j.jmb.2016.04.025 (PMC4942838; doi:10.1016/j.jmb.2016.04.025)
Supplement: Supplementary file 1 — Supplementary material [file mmc1.pdf]

SUPPLEMENTARY INFORMATION

The Nucleosome Remodeling and Deacetylase Complex NuRD is built from preformed catalytically active sub-modules.

W. Zhang[a1], A. Aubert[b1], J. M. Gomez de Segura[b], M. Karuppasamy[b], S. Basu[a], A. Murthy[a], A. Diamante[a], T. A. Drury[a], J. Balmer[a], J. Cramard[d], A. A. Watson[a], D. Lando[a], S. F. Lee[e], M. Palayret[e], S. Kloet[f], A. Smits[f], M. Deery[g], M. Vermeulen[f], B. Hendrich[d], D. Klenerman[e], C. Schaffitzel[bc2], I. Berger[bc2], and E. D. Laue[a2]

Affiliations:

[a] Department of Biochemistry, University of Cambridge, 80 Tennis Court Road, Cambridge CB2 1GA, United Kingdom

[b] EMBL Grenoble, 71 avenue des Martyrs, CS 90181, 38042 Grenoble Cedex 9, France

[c] The School of Biochemistry, University of Bristol, University Walk, Clifton, BS8 1TD, United Kingdom

[d] Wellcome Trust – Medical Research Council Stem Cell Institute, Gleeson Building, University of Cambridge, Tennis Court Road, Cambridge CB2 1QR

[e] Department of Chemistry, University of Cambridge, Lensfield Road, Cambridge CB2 1EW, United Kingdom

[f] Department of Molecular Biology, Radboud Institute of Molecular Life Sciences, M850/3.79 Geert Grooteplein Zuid 30, 6525 GA Nijmegen, The Netherlands

[g] Cambridge Centre for Proteomics, Cambridge System Biology Centre, Wellcome Trust Stem Cell building, University of Cambridge, Department of Biochemistry, Tennis Court Road, Cambridge, CB2 1QR

[1] These authors contributed equally to this work.

[2] To whom correspondence should be addressed:

SI METHODS

NuRD complex preparation from endogenous source material

We modified a Gateway destination plasmid for metallothionein promoter driven protein expression in *Drosophila melanogaster* S2 Schneider cells. The Gateway plasmid containing an N-terminal GFP-tag[1] was modified by introducing a DNA sequence encoding two TEV protease cleavage sites followed by a deca-histidine tag in frame with the C-terminus of the GFP, giving rise to a GFP-2xTEV-10xHis tag preceding the target gene insertion site. The Quick-change Lightning site-directed mutagenesis kit (Agilent Technology) and a set of oligonucleotide primers (Supplementary Table I) were utilized. The gene encoding for the bait was inserted into the pDONR221 plasmid, giving rise to a Gateway entry clone. The sequence verified construct was then recombined into the modified destination vector according to the manufacturer's protocols (Thermo Fischer Scientific).

Destination plasmids expressing GFP-tag (as a control) and GFP-2xTEV-10xHis-tagged protein, respectively, were transformed into S2 Schneider cells (Thermo Fisher Scientific). Stable cell lines were selected by adding 20 µg/mL blasticidin (Thermo Fisher Scientific). Transformed cell cultures were grown in ExpressFiveSFM medium supplemented with 2mM Glutamine (Thermo Fisher Scientific) at 25°C in tissue culture (GBO) or Erlenmeyer flasks orbiting at 80 rpm (Corning). At a density of 8 million cells/ml, cultures were induced by adding $CuSO4$ (to 0.1 mM, so as to express near endogenous levels of the GFP-tagged protein as determined by Western blot) and incubated for 18 hours.

Cells were pelleted, lysed and complexes prepared for mass spectrometry as described previously[2]. For analysis by electron microscopy, the protocol was modified as follows. Nuclear extracts were incubated for 2h with GFP-Trap beads (ChromoTek) at a ratio of 3 mg total protein to each 10 µL aliquot of beads at 4^0C in Extraction Buffer [20 mM Hepes pH 7.5, 420 mM NaCl, 20% v/v glycerol, 0.1% NP40, 0.5 mM DTT, and protease inhibitors (0.1 mM PMSF, 10 µg/mL Leupeptin, and 10 µg/mL Pepstatin)]. Following incubation, the beads were collected and washed

three times with Washing Buffer (Extraction Buffer supplemented with 300 mM NaCl), followed by two washes with TEV Digestion Buffer (50 mM Hepes pH 7.5, 200 mM NaCl, 2% v/v glycerol, and 0.5 mM DTT). Captured protein was released from the beads by addition of 2 μg of purified TEV protease. The eluted complex was further purified by gradient centrifugation (Grafix) in the presence of mild crosslinking [3]. About 100 to 200 μL complex (70 – 100 μg) was loaded onto a centrifuge tube (Beckman 7/16x2 – 3/8 P.A) containing a gradient of 10-30% v/v glycerol and 0-0.15% glutaraldehyde in Grafix Buffer (50 mM HEPES pH 7.5, 150 mM NaCl, and 0.5 mM CHAPS) generated with a Gradient Master device (Wolf Laboratories) and centrifuged at 4^0C, 34,000 rpm for 18 h in a SW60Ti rotor (Beckman). Centrifuged samples were fractionated into 0.2 mL aliquots using a peristaltic pump. The cross-linking activity of glutaraldehyde was quenched with 80 mM glycine pH 7.6 immediately after fractionation and the cross-linking state of the complexes was verified by SDS-PAGE using NuPAGE 4-12% Bis-Tris gradient gels (Novex) in MOPS-SDS running buffer followed by staining with Instant blue (Exedeon).

Preparation of recombinant NuRD complexes PMR and PMMR

Recombinant NuRD complexes were expressed using the MultiBac baculovirus insect cell expression system[4]. Synthetic genes (Genscript) encoding p55, MTA-like and Rpd3 were inserted into the Acceptor plasmid pKL using the Multiplication Module[4] giving rise to transfer plasmid pKL_PMR. MTA-like was expressed with an N-terminal 10xHistidine – 3xFLAG 1xTEV tandem affinity purification tag. The gene encoding for maltose binding protein (MBP) tagged MBD-like protein was inserted into pSPL to yield plasmid pSPL_MBD-like. Cre-LoxP mediated plasmid fusion[4] of pSPL_MBD-like and pKL_PMR resulted in transfer plasmid pLox_PMMR. Transfer plasmids pKL_PMR and pLox_PMMR, respectively, were transformed into DH10EMBacY cells containing the EMBacY baculoviral genome. Composite baculoviruses were generated in DH10EMBacY cells by Tn7 transpositions and blue/white selection, and virus was prepared following standard protocols[4]. Protein complexes were expressed in Sf21 insect cell cultures

infected with the composite baculovirses. Cell pellets were stored at -80°C.

Cell pellets were resuspended in two bed volumes of Buffer A (10 mM Hepes pH 7.5, 1.5 mM $MgCl_2$, 10 mM KCl, 0.1% NP-40, and 0.5 mM DTT) freshly supplemented with 0.1 mM PMSF, 10 μg/mL Leupeptin (Sigma) and 10 μg/mL Pepstatin A (Sigma). Following 20 minutes incubation on ice, cells were lysed by homogenization using a type B (tight) dounce homogenizer for 30 strokes. Nuclei were separated from the cytoplasmic extract by centrifugation at 1700 x g for 15 minutes at 4°C. Cleared cytoplasmic extracts were subjected to Flag-tag affinity purification using anti-FLAG M2 affinity gel (Sigma, pre-equilibrated in modified Buffer A' containing 150 mM NaCl and 10% glycerol) by incubation at 4°C for 1.5 hours on a rolling wheel. Following extensive washing (>10 bed volumes), bound proteins were eluted with a 3xFLAG peptide according to the manufacturer's protocols (Sigma). The purity and quantity of the eluted proteins were evaluated on an Instant Blue (Expedeon) stained 4-12% Bis-Tris gel (Novex). Subsequently, the complexes were further purified by size exclusion chromatography (SEC) on a Superose 6 Increase 3.2/300 column (GE Healthcare Life Sciences) in SEC Buffer (50 mM Hepes pH 7.5, 150 mM NaCl, and 2% glycerol).

Recombinant co-expression of all seven NuRD subunits using MultiBac

Recombinant co-expression of all known components of the Drosophila NuRD complex was carried out by the MultiBac system[4]. The same expression construct pKL-PMR as for expressing PMR and PMMR complexes was used. Plasmid pKL-PMR was combined with a pSPL plasmid into which MBD-like tagged with MBP at its N-terminus and CHD4 like, each in a separate expression cassette, had been inserted. A construct containing to independent expression cassettes for both Simjang with an N-terminal G18292 which is thought to encode the *Drosophila* Doc1 homologue was prepared by inserting the corresponding synthetic genes (Genscript) into the MultiBac plasmid pUDCM. pLox-dNuRD containing genes encoding for a full complement of dNuRD subunits was generated by Cre-LoxP mediated plasmid fusion[4]. pLox-dNuRD was inserted into the EMBacY baculoviral genome, virus amplified and proteins expressed using

standard protocols[4]. Protein complex purification followed the protocol used for purifying PMR and PMMR complexes.

Size-exclusion chromatography coupled to multi-angle laser light scattering (SEC-MALLS) analysis of PMMR complex.

SEC-MALLS of the PMMR complex was performed using a HPLC system (LaChrom Elite, VWR, Radnor, Pennsylvania, USA) and a Superose 6 10/30 GL column (GE Healthcare Life Sciences, Pittsburgh, Pennsylvania, USA) equilibrated in SEC-MALLS Buffer (50mM Tris pH 8.5, 150mM NaCl, 0.5mM TCEP). Samples were injected at room temperature (injection volume: 50uL) at a concentration ranging from 0.98 mg/mL to 2.77 mg/mL. Eluent was pumped at a flow-rate of 0.4-0.5mL/min and monitored using DAWN Heleos-II multi-angle light scattering detector and an Optilab T-rEX refractometer (both instrument from WYATT Technology, Santa Barbara, California, USA). Signal from the light scattering photometer and the refractometer were captured and analyzed using the dedicated ASTRA software provided by the manufacturer. The analysis by SEC-MALLS indicated a weight-averaged molecular mass of 596.6±7 kDa for PMMR. The molecular mass determined by SEC-MALLS was constant throughout the chromatographic peak indicating that the complex was monodisperse (M_w/M_n=1.00±0.01).

Electrophoretic mobility shift assays

Synthetic DNA (Genscript) encoding for helicase deficient mutant CHD4K757R and an N-terminal decahistidine-triple FLAG epitope tag was inserted into plasmid pFL and expressed with the MultiBac system (1). Recombinant PMR, PMMR (75 $\mu g.mL^{-1}$) and CHD4K757R (325 nM) were mixed with mononucleosomes (30 nM) in 20 µL final volume Incubation Buffer (50 mM Tris, 150 mM KCl, 4 mM $MgCl_2$, 0.4 mM EDTA, 1 mM DTT, 5 % glycerol, pH 7.5). Reactions were incubated 30 min at room temperature before loading on a 20 x 20 cm 5 % polyacrylamide, 0.2x TBE gel. Electrophoresis was performed for 10 h at 100 V and 4 °C. Polyacrylamid gels were

stained by SYBR Gold (Thermo Fisher Scientific, Waltham, Massachussets USA) according to manufacturer's recommendation and imaged by recording fluorescence at 473 nm using a Typhoon fluorimager (GE Healthcare Lifesciences Pittsburgh, Pennsylvania, USA).

Generation of mouse embryonic stem cells (mES) expressing mEos3-tagged CHD4

Mouse embryonic stem (mES) cells expressing CHD4 tagged at the C-terminus with mEos3 were generated by knock-in of a cassette containing mEos3 and a puromycin selection gene into one CHD4 allele in an $MBD3^{Flox/-}$ mES cell line[5] using CRISPR/Cas9 and a guide RNA targeting exon 39 of the gene. The puromycin cassette was then removed using Dre recombinase to generate the endogenous CHD4 allele with a C-terminal mEos3 fusion. To convert this line to MBD3-null cells the remaining MBD3 allele was then knocked out using Cre recombinase. Wild-type and MBD3-null mES cells expressing the mEos3 tagged CHD4 were cultured in standard serum and LIF conditions on 35 mm glass bottom culture dishes (MatTek Corp.)[6].

SI FIGURES

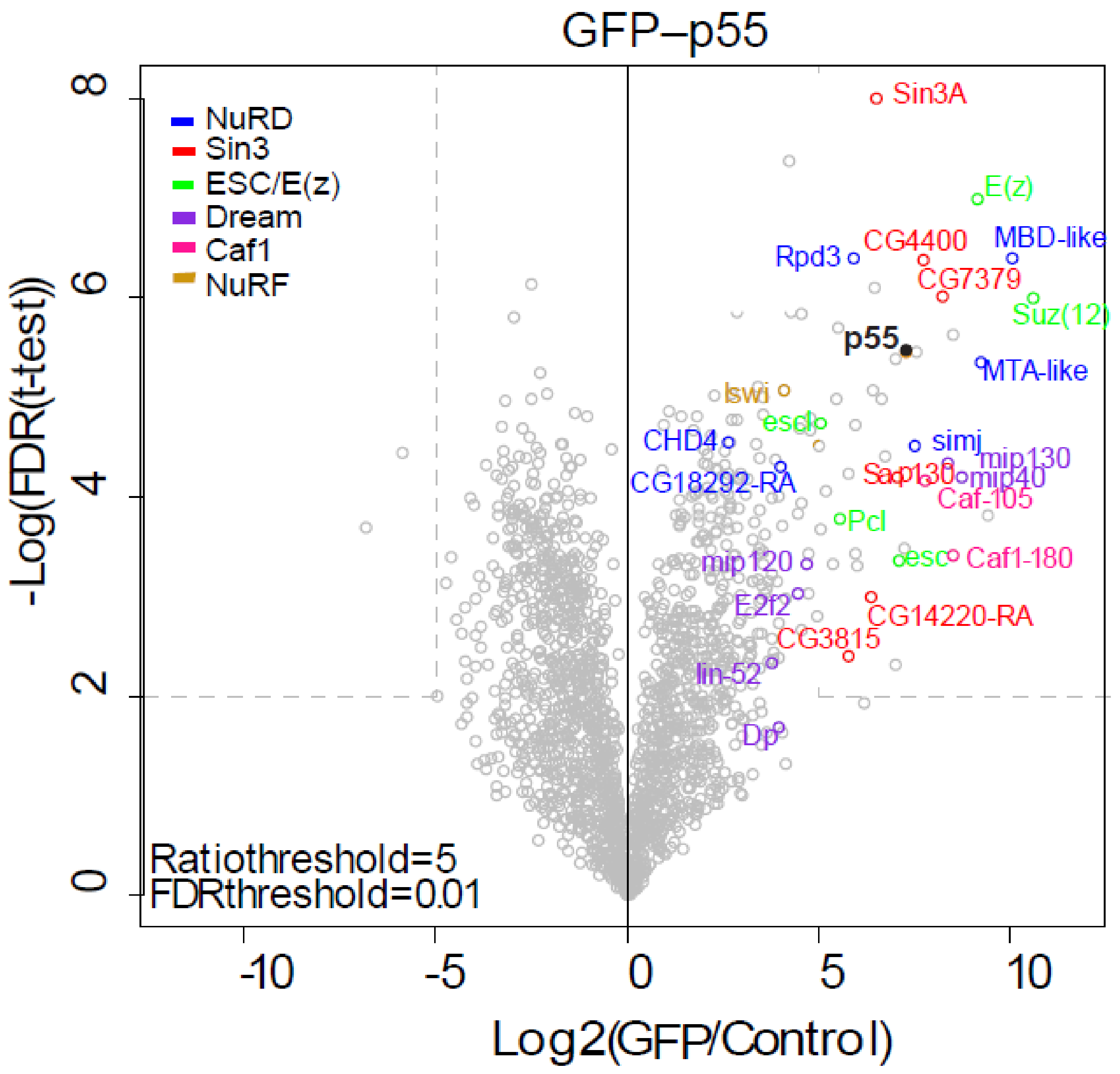

Supplementary Figure S1: Annotated Volcano plot of GFP-p55 affinity capture. Statistically enriched proteins in the GFP-p55 pull-down experiment using GFP-p55 were identified by permutation-based FDR-corrected t-test, and are shown in a Volcano plot. CG18292 protein is consistently enriched supporting CG18292, a *Drosophila melanogaster* Doc1 protein[7]. Components from remodeling complexes different from NuRD which contain p55 are likewise detected (color code in inset, top left).

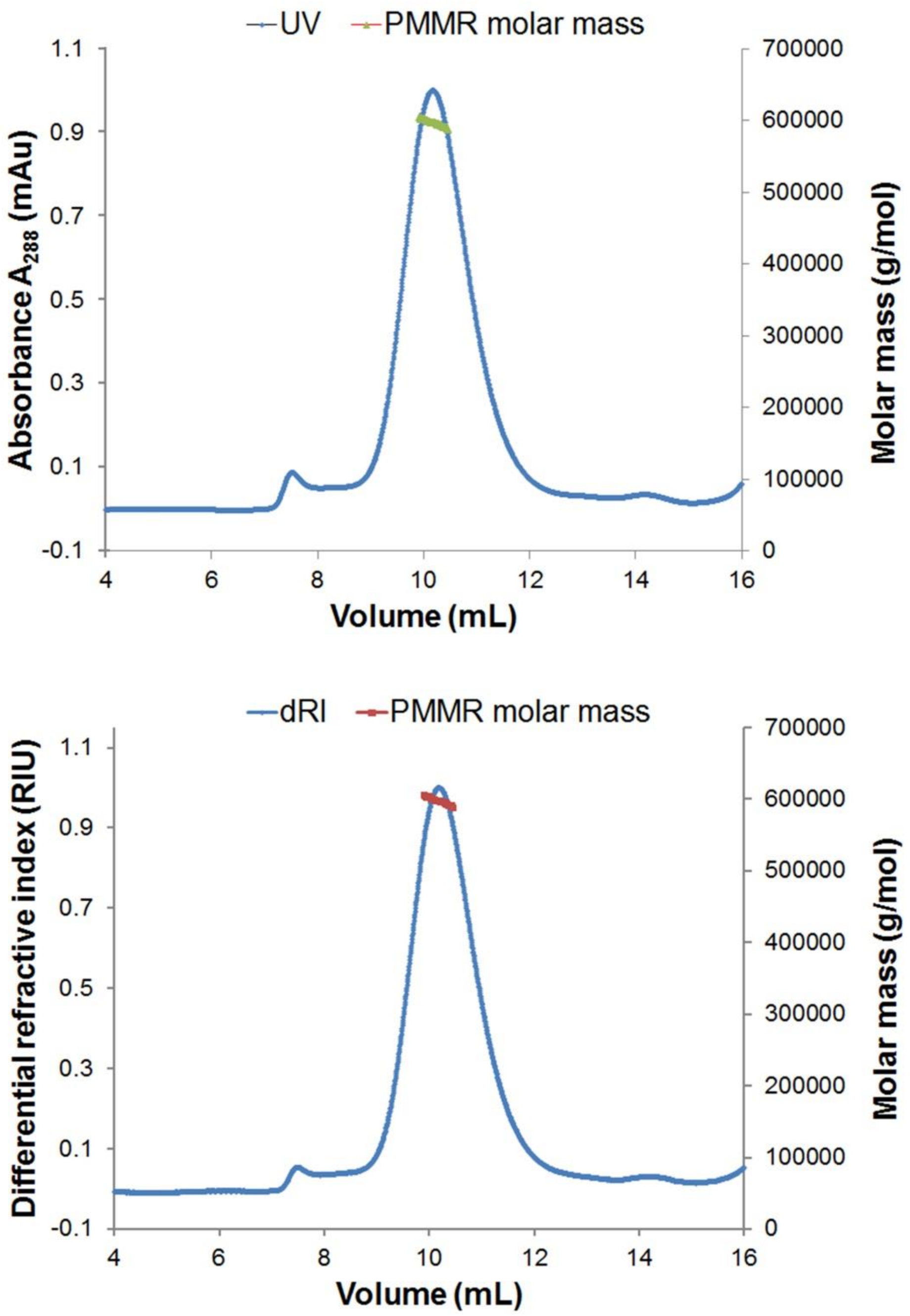

Supplementary Figure S2: SEC-MALS of recombinant PMMR complex. Purified PMMR was analysed by multi-angle light scattering (MALS) following size exclusion chromatography (SEC). SEC elution profile for PMMR is shown measured by UV (top) and interference (bottom). Both analyses evidence molecular weights of 600 kDa for PMMR complex. This is consistent with subunit stoichiometries of 4:2:2:1 for p55, MTA-like, Rpd3, MBD-like respectively in good agreement with analyses by mass spectroscopy (Fig. 1d).

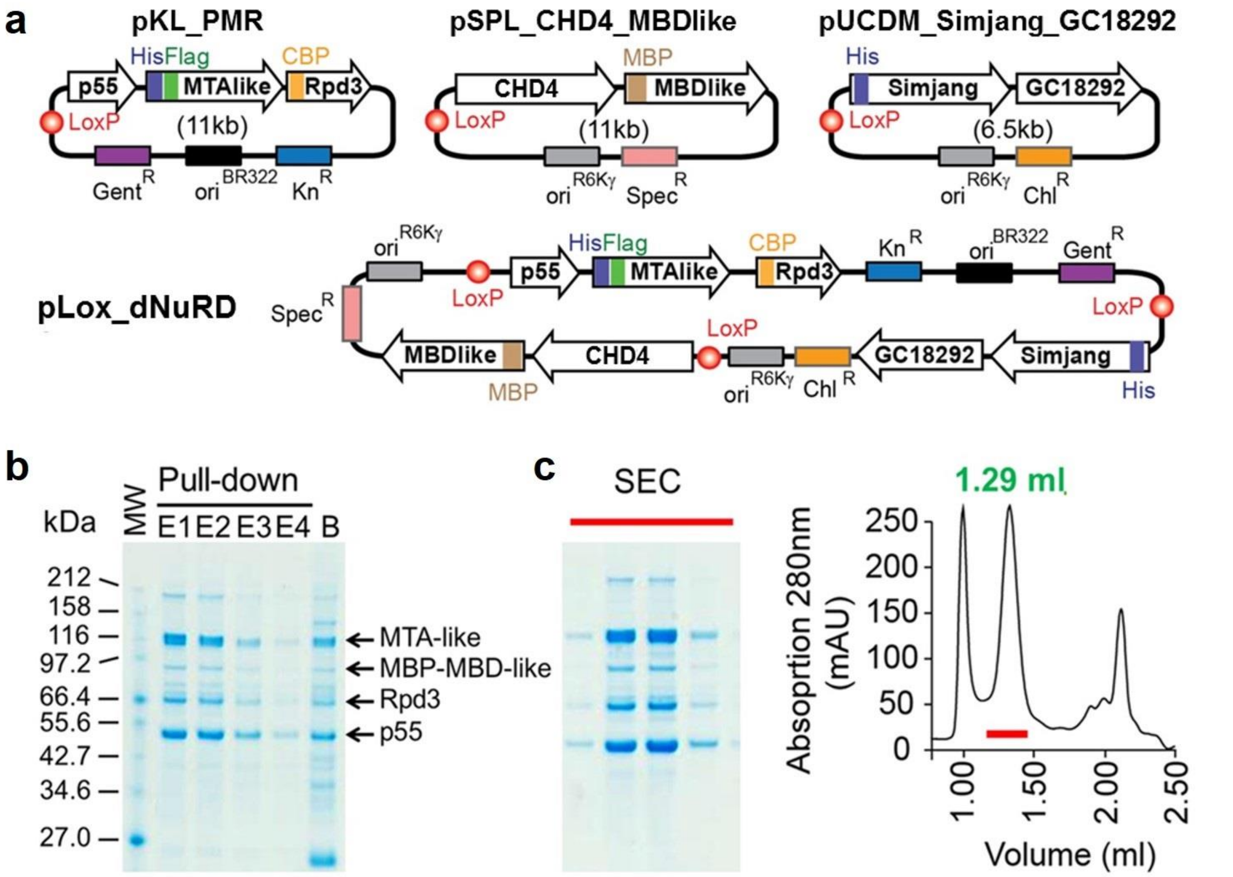

Supplementary Figure S3: MultiBac co-production of full complement of NuRD subunits.

(a) Constructs for co-expressing all seven known NuRD subunits in the MultiBac system [4] are shown in a schematic representation. pKL, pSPL and pUCDM and MultiBac Acceptor and Donor plasmids, respectively[4], LoxP sites used for Cre recombination are shown as circles filled in red. Resistance markers and origins of replication are depicted as colored boxes. Genes encoding for NuRD subunits are shown as arrows filled in white. PMR stands for p55, MTA-like and MBD-like, respectively. GC18292 is the putative *Drosophila* Doc1 subunit[7]. Tags are shown as colored rectangles and marked. HisFlag, tandem affinity purification tag comprising a hexahistidine tag and a triple FLAG epitope; MBP, maltose binding protein; CBP, calmodulin binding peptide. Gent, gentamycin resistance marker; Kn, kanamycin resistance marker; Spec, spectinomycin resistance marker, Chl, chloramphenicol resistance marker. Origins or replication (ori^{BR322} for pKL, $ori^{R6K\gamma}$ for pSPL and pUCDM are indicated. pLox_dNuRD is generated from pKL_PMR, pSPL_CHD4_MBDlike and pUCDM_Simjang_GC18292 by Cre-LoxP mediated multi-plasmid

fusion. PMMR contains in addition to PMR a gene encoding for MBP-tagged MBD-like protein**.** The size of the plasmids (in kilobases, kb) is indicated in brackets**.** **(b)** Recombinant complex was enriched from cell lysate by FLAG affinity purification. SDS-PAGE sections of the eluted complex are shown. E1-3, elution fractions; B, affinity beads; MW, molecular weight marker. Molecular weights of marker bands are indicated in kDa. Predominantly MTA-like, MBD-like, Rpd3 and p55 are eluted (corresponding to a PMMR complex). The high molecular weight band at approximately 200 kDa corresponds to MTA-like protein, presumably by dimerization in SDS-PAGE. **(c)** Size exclusion chromatography (SEC) of affinity-purified recombinant complex from the pLox_dNuRD expression is depicted. The SEC profile (right) evidences two peaks, one of which (marked by red bar) contains NuRD subunits evidenced by the corresponding SDS-PAGE sections (left). Peak retention volume of the complex purified from expressing pLox_dNuRD is 1.29 ml (green), consistent with the elution volume of the complex purified from expressing pKL_PMMR (Fig. 2, Supplementary Fig S2).

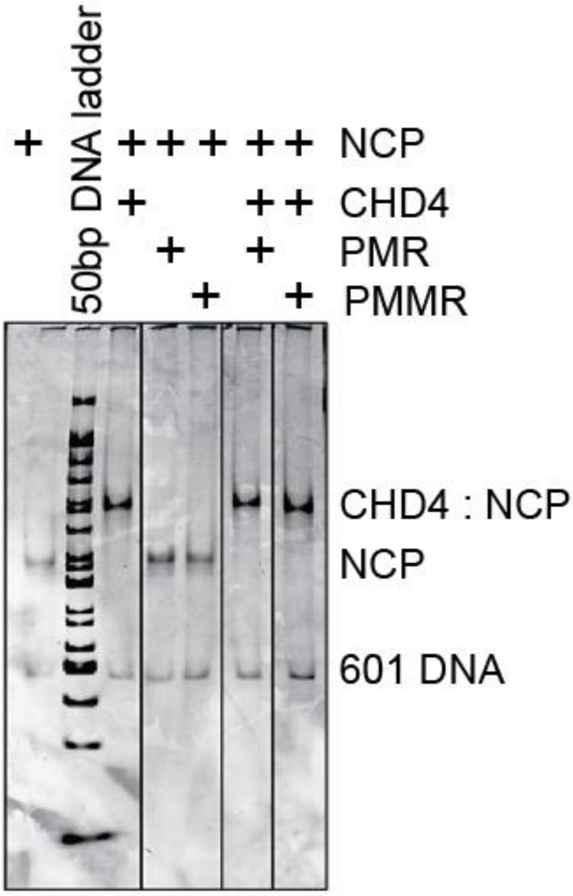

Supplementary Figure S4: Nucleosome-binding activity of NuRD subunits. NuRD subcomplexes PMR and PMMR, and a helicase defective mutant of the catalytic subunit CHD4 (CHD4K757R) were assayed by electrophoretic mobility shift assay to assess binding to a recombinant mono-nucleosome core particle (NCP) containing the 601 nucleosome positioning sequence[8]. Neither PMR nor PMMR were observed to stably interact with the NCP in the EMSA, whereas CHD4K757R formed a stable complex with the nucleosomal substrate. This shifted band could not be super-shifted by the addition of PMR or PMMR, suggesting that a stable interaction between CHD4K757R and PMR or PMMR may critically depend on the presence of Simjang and/or CG18292.

SI TABLE

**Supplementary Table I:** Primers used in this study to generate GFP-bait construct.		
**Purpose**	**Primer Name**	**Sequences**
Insert TEV after GFP	pMTGfpTev_F	5'-atg gac gag ctg tac aag agc gaa aac ctg tat ttt cag ggc ccc gga ggc gga ggc gga ctg atc-3'
	pMTGfpTev_R	5'-gat cag tcc gcc tcc gcc tcc ggg gcc ctg aaa ata cag gtt ttc gct ctt gta cag ctc gtc cat-3'
Insert 6x His after TEV	pMTGfp6His_F	5'-aac ctg tat ttt cag ggc cat cac cat cac cat cat ccc gga ggc gga ggc gga-3'
	pMTGfp6His_R	5'- tcc gcc tcc gcc tcc ggg atg atg gtg atg gtg atg gcc ctg aaa ata cag gtt-3'
Insert 2nd TEV before 6xHis	pMTGfpTev2_F	5'-aac ctg tat ttt cag ggc gag aat cta tac ttc caa gga cat cac cat cac cat cat-3'
	pMTGfpTev2_R	5'-atg atg gtg atg gtg atg tcc ttg gaa gta tag att ctc gcc ctg aaa ata cag gtt-3'
Insert 4 extra His for a 10xHis tag	pMTGfp10His_F	5'-aac ctg tat ttt cag ggc cat cac cat cac cat cat cac cat cat cac ccc gga ggc gga ggc gga-3'
	pMTGfp10His_R	5'-tcc gcc tcc tcc tcc ggg gtg atg atg gtg atg atg gtg atg gtg atg gcc ctg aaa ata cag gtt-3'

SI REFERENCES:

1. D'Avino, P. P., Archambault, V., Przewloka, M. R., Zhang, W., Laue, E. D. & Glover, D. M. (2009). Isolation of protein complexes involved in mitosis and cytokinesis from Drosophila cultured cells. *Methods Mol Biol* **545**, 99-112.
2. Smits, A. H., Jansen, P. W., Poser, I., Hyman, A. A. & Vermeulen, M. (2013). Stoichiometry of chromatin-associated protein complexes revealed by label-free quantitative mass spectrometry-based proteomics. *Nucleic Acids Res* **41**, e28.
3. Stark, H. (2010). GraFix: stabilization of fragile macromolecular complexes for single particle cryo-EM. *Methods Enzymol* **481**, 109-26.
4. Fitzgerald, D. J., Berger, P., Schaffitzel, C., Yamada, K., Richmond, T. J. & Berger, I. (2006). Protein complex expression by using multigene baculoviral vectors. *Nat Methods* **3**, 1021-32.
5. Kaji, K., Caballero, I. M., MacLeod, R., Nichols, J., Wilson, V. A. & Hendrich, B. (2006). The NuRD component Mbd3 is required for pluripotency of embryonic stem cells. *Nat Cell Biol* **8**, 285-92.
6. Palayret, M., Armes, H., Basu, S., Watson, A. T., Herbert, A., Lando, D., Etheridge, T. J., Endesfelder, U., Heilemann, M., Laue, E., Carr, A. M., Klenerman, D. & Lee, S. F. (2015). Virtual-'light-sheet' single-molecule localisation microscopy enables quantitative optical sectioning for super-resolution imaging. *PLoS One* **10**, e0125438.
7. Reddy, B. A., Bajpe, P. K., Bassett, A., Moshkin, Y. M., Kozhevnikova, E., Bezstarosti, K., Demmers, J. A., Travers, A. A. & Verrijzer, C. P. (2010). Drosophila transcription factor Tramtrack69 binds MEP1 to recruit the chromatin remodeler NuRD. *Mol Cell Biol* **30**, 5234-44.
8. Lowary, P. T. & Widom, J. (1998). New DNA sequence rules for high affinity binding to histone octamer and sequence-directed nucleosome positioning. *J Mol Biol* **276**, 19-42.
